# An examination of the psychological mechanisms underlying the social existence and cognitive investment of college students in an online learning environment

**DOI:** 10.3389/fpsyg.2026.1792669

**Published:** 2026-05-13

**Authors:** Xuemin Shang, Aimin Tang

**Affiliations:** Faculty of Education, Qufu Normal University, Qufu, China

**Keywords:** cognitive engagement, online learning, perceived interactivity, psychological mechanism, social presence

## Abstract

Addressing the common challenges in online education, where the body is present but the mind is disengaged and deep learning is limited, it is crucial to examine how social interactions in virtual environments translate into intrinsic cognitive motivation. This study aimed to explore the mechanism linking social presence (SP) and cognitive engagement (CE) in online learning environments, with perceived interactivity (PI) introduced as a key variable for empirical testing. A total of 500 university students with online learning experience were recruited. Data were collected using the Community of Inquiry scale, the Online Learning Engagement scale, and the PI scale. Correlation and hierarchical regression analyses were conducted using SPSS software. Results indicated that SP positively and robustly predicted CE (*β* = 0.582, *p* < 0.001). This finding suggests that, in physically separated learning contexts, learners’ perceptions of interpersonal authenticity and group belonging effectively predict their participation in higher-order cognitive activities. PI was highly correlated with SP but did not show a significant moderating effect between SP and CE. The study confirmed that high-quality interactive experiences and SP were essential conditions for fostering deep learning online. Enhancing multidimensional interaction design to strengthen learners’ SP is a viable strategy to promote deep cognitive processing and improve the quality of online instruction.

## 1 Introduction

With the wide application of information and communication technology, online education has become the core component of higher education system. However, the physical separation often destroys the emotional connection between teachers and students and between peers, resulting in the learning effect of many online courses staying in the shallow layer, which is manifested as “although people are there, their hearts are not there” (Luo and Sun, 2025). Existing research mainly focuses on optimizing technical conditions, such as network bandwidth and platform functions, or analyzing observable behavioral indicators, such as click times and login duration. However, these studies pay little attention to the hidden and complicated psychological processes that occur in these technical environments (Shen et al., 2024). This gap makes it difficult to explain why there are still significant differences in learners’ deep learning achievements under the same technical conditions (Luan et al., 2023).

Social presence (SP) is the core dimension of the framework of Community of Inquiry (CoI), and it is considered as a bridge between virtual environment and real psychological experience. It refers to the degree to which participants perceive others as “real people” in media communication (Miao et al., 2022). Although previous studies have confirmed that SP has a positive impact on learning satisfaction, the mechanism of social participation driving higher-order cognitive processing, especially how it translates into cognitive engagement (CE), has not been fully explained (Pan, 2023). Cognitive engagement reflects learners’ psychological efforts and their use of deep learning strategies, making it a key indicator of online learning quality (You, 2022). Therefore, it is very important to clarify the internal path from social participation to cognitive input, both theoretically and practically, to improve the online teaching effect (Adeshola and Agoyi, 2023).

Existing research mainly focuses on optimizing technical conditions (such as network bandwidth) or analyzing observable behavioral indicators (such as clicks). Few studies have explored the complex psychological process behind the technological environment. Under the CoI framework, social participation is regarded as a bridge between virtual environment and real psychological experience. Previous studies have proved that it has a positive predictive effect on learning satisfaction; however, the internal logic of social participation driving higher-order cognitive processing-that is, the process of transforming it into cognitive input-has not been fully explained. This study reconstructs the relationship between technological environment and deep learning by constructing a psychological interaction model including perceptual interaction (PI), SP and CE. Different from the one-way causal path assumed in the past, this study focuses on how perceived interaction, as an environmental variable, can predict cognitive experience together with the perception process, and empirically tests its potential boundary adjustment effect.

## 2 Literature review

### 2.1 Definition and significance of SP

Under the framework of CoI, SP is defined as the degree to which learners perceive the “truthfulness” of their peers and teachers in media communication. Its core performance includes open communication, emotional expression and group cohesion. In online learning, which lacks a shared physical environment, SP plays a psychological role by organizing scattered individuals into learning communities. It affects whether learners are willing to express their views, disclose difficulties and participate in the construction of collaborative meaning. Empirical studies on different courses and learners groups have consistently confirmed the relationship between SP and learning engagement, satisfaction and learning outcomes, and these findings are also applicable to online learning samples of universities (Wang, 2022; ElSayad, 2024). The evidence at the level of teaching strategies shows that increasing visibility clues, strengthening peer interaction rules and providing emotional feedback can enhance SP and promote higher quality learning participation (Xia et al., 2024; Garrels and Zemliansky, 2022). Studies in specific situations (such as language learning) further show that SP is not only related to engagement, but also may play an intermediary role between individual psychological resources and learning behavior (Gao et al., 2024).

Recent studies have expanded the evidence base of SP from a single course survey to more extensive and comprehensive data. Meta-analysis shows that various forms of presence within the framework of learning community are stably related to learning achievement and satisfaction. The relationship between student participation and academic performance can be repeatedly verified under different research designs and sample conditions (Adam et al., 2025; Izatullah et al., 2026). In addition, evidence of process orientation has emerged, providing a more detailed path. The researchers combined the discussion interaction structure with the course process data, and used social network analysis indicators to quantify the formation and fluctuation of student participation, which went beyond the description based on the perception of the end of the course (Norz et al., 2024). The study under the condition of emergency online teaching shows that even in uncertain learning environment, student participation can still explain the difference between participation and learning benefits, which highlights its continuous importance to curriculum stability and learning resilience (Li et al., 2023).

### 2.2 Definition and importance of CE

Learning input is usually regarded as a multidimensional structure. CE emphasizes learners’ psychological efforts and the intensity of strategies adopted to understand and master the learning content. It focuses on deep processing, self-regulation, knowledge refining and migration. In the online learning environment, the study based on behavior log and discussion text analysis shows that deep learning strategies and intrinsic motivation are more conducive to maintaining learning engagement. Higher-order cognitive processing in discussion is closely related to learning achievement and continuous participation (Li et al., 2022). If input is only regarded as a comprehensive index, the influence of SP on “deep cognitive activities” may be weakened, which makes it difficult to explain the persistent differences of learners’ cognitive input under the same technical conditions (Liu et al., 2023).

Recent discussions on cognitive input emphasize its performance in the process of task promotion and interactive consultation. The study began to use discussion text, learning trajectory data and automatic identification methods to describe cognitive input. Systematic evaluation shows that enhancing cognitive input depends on continuous high-quality discussion and effective guidance; Increasing the frequency of interaction does not necessarily translate into deep cognitive processing (Moore and Miller, 2022). The research of MOOC shows that learners’ expression, response and higher-level cognitive components in the discussion forum can form a variety of participation modes, which correspond to different learning outcomes. Therefore, CE is best understood as a learning state produced by the coupling of various elements (Liu et al., 2024). The longitudinal study from synchronous online learning further reveals that the influence of learning readiness and participation on satisfaction will evolve with time. In the later stage of the course, participation plays a more important role in shaping learners’ experience, and the formation of cognitive experience also presents stage characteristics (Ji et al., 2022).

### 2.3 Definition, dimensions, and role of PI in online learning

PI emphasizes learners’ subjective experience of the interactive process and distinguishes it from the objective existence of platform functions. PI is usually conceptualized as a multi-dimensional structure, including controllability, responsiveness, two-way communication and interactive fun. These experiences will affect immersion, psychological belonging and willingness to continue using the platform (Joo and Yang, 2023). The research in dialogue agent and human-computer interaction environment shows that there is a stable relationship among PI, SP, trust and willingness to continue using, which indicates that interactive experience can change psychological state through the availability and interpretability of social cues (Zhang et al., 2025). Most existing studies focus on the results of PI in terms of satisfaction, trust or willingness to use, but there are limited opinions on whether PI is related to CE in formal online courses (Hong and Satjawathee, 2024).

Recent studies have emphasized two development trends that make PI more closely related to learning situations. First of all, PI is increasingly placed in the chain of “teaching support → learner’s behavior → learning achievement”. Empirical research shows that teacher and peer support can promote deeper learning participation through learners’ behavioral engagement, and PI can be used as a comprehensive evaluation of learners’ interactive function (Kuo et al., 2013). Secondly, with the increasing popularity of artificial intelligence (AI) teaching agents and learners’ interaction with AI, the measurement of PI has also begun to focus on the interaction between learners and AI. The scale development research has subdivided the PI in the interaction between learners and AI into the dimensions of responsiveness, personalization, sense of control and process continuity, which provides an operational basis for testing PI, learning participation and learning experience in the same model (Ilieva et al., 2026). Related research has also observed stable links between learners’ judgments of AI instructor effectiveness and their engagement, motivation, and learning experience. This indicates that technological interaction is no longer merely an antecedent of usage intention but has begun to enter explanations of learning engagement mechanisms (Al Harrasi et al., 2025).

### 2.4 Clues on the relationships among SP, PI, and CE and research gaps

Existing evidence indicates a stable relationship between SP and CE, and a strong theoretical coupling between PI and SP. Conceptually, PI provides learners with an experience of interaction affordances, increasing the visibility and immediacy of social cues, reducing communication costs, and facilitating judgments of others’ real presence. Once SP is enhanced, learners are more likely to engage in activities such as expressing opinions, negotiating interpretations, and co-constructing knowledge, thereby elevating CE levels. While prior CoI-based literature has established the value of SP, discussions of PI have largely remained at the level of technical experience and behavioral intention. Few studies have systematically analyzed SP, PI, and CE within a single testable model. This gap leaves unanswered practical questions in online course design: when interaction features are increased, which psychological mechanisms transform interactive experiences into deep CE?

Recent evidence highlights three relationship clues. First of all, the relationship between social participation and learning engagement is not limited to the single sample; Interdisciplinary research shows that SP’s predictive effect on learning outcomes is consistent in different fields, and participation can be used as a direct measure of “active learning performance” (Lim and Richardson, 2021; Edwards and Taasoobshirazi, 2022). Secondly, when examining the interactive experience in real classroom situations, the evidence is no longer limited to the platform function of self-report. The study of synchronous online learning shows that it significantly affects classroom participation behavior, and the continuity and reaction rhythm of interactive process are positively correlated with participation (Jarrah et al., 2025). Thirdly, the latest structural equation model under the CoI framework began to distinguish participation dimensions more accurately. The existence of three types has different effects on participation, which shows that it is explanatory to include PI as a boundary condition in the model (Na et al., 2024; Alshammari and Alrehaili, 2025). Despite these advances, there is still a lack of empirical tests to integrate PI, SP and CE into a single model to clarify how interactive experience can be transformed into deep cognitive processing. Therefore, the decision-making of curriculum design still lacks solid theoretical foundation and transferable guidance.

## 3 Theoretical framework and research hypotheses

### 3.1 Theoretical framework

The CoI framework holds that teaching telepresence, SP and cognitive telepresence jointly support the deep learning experience in online education. Meta-analysis evidence shows that these three kinds of telepresence have a stable positive correlation with learning achievement, satisfaction and participation, which provides an empirical basis for applying this framework to online courses in higher education (Martin et al., 2022). Under this framework, this study takes CE as the result variable, SP as the key psychological antecedent, and situational awareness (PI) as the situational empowerment variable. In this study, the SP-CE causal chain is constructed, and the boundary conditions of PI are investigated.

The evidence provided by recent CoI research is especially suitable for defining the path in this study. Meta-analysis and systematic evaluation support the stability of the relationship of “presence → learning achievement” from a comprehensive level (Mykota, 2025). The study of structural equation model has linked the variables of telepresence with learners’ self-regulation and learning strategies, which proves that there is a testable chain relationship between telepresence and learning process variables. These findings provide a practical interface for bringing PI into the mechanism chain and positioning it as a boundary condition. The study carried out during online emergency teaching and online post-epidemic courses reached a similar conclusion: variables related to learning communities can explain the differences in participation and learning outcomes. This shows that the framework of community participation has universal applicability under various online teaching conditions (Regnier et al., 2024).

**Figure 1:**
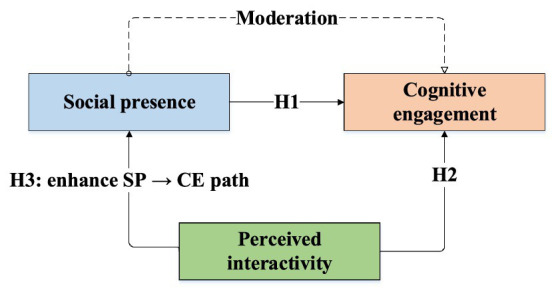
Hypothesized model of SP, PI, and CE.

Figure 1 shows the hypothetical relationship model among SP, PI and CE.

### 3.2 SP and CE

In the online environment, whether learners can carry out high-level CE depends on whether their attention resources are allocated to content understanding and strategy application. When SP is strengthened, learners will form stable expectations for the authenticity and responsiveness of communication partners, thus reducing the cost of expression and promoting the exchange of opinions and explanatory consultation. This process increases the proportion of cognitive resources related to tasks, and changes learning activities from passive information reception to meaning construction and knowledge integration (Jiang and Yu, 2025). CoI research across different courses and samples consistently shows that there is a positive correlation between SP and learning participation and learning outcomes, and this relationship is still robust in the online learning environment of higher education (Abidin et al., 2023; Zhou et al., 2025). Based on this, this study proposes:

*H1*: SP has a significant positive effect on CE.

### 3.3 PI and CE

PI reflects learners’ sense of control, timeliness of feedback and fluency of two-way communication in the process of interaction. When this experience is improved, learners are more likely to get timely feedback and clarification opportunities, so that they can adjust their strategies and self-monitor more effectively, thus promoting deep processing. Educational technology research shows that PI is a more direct predictor of learning motivation and behavior than platform function itself. In a highly interactive environment, learners tend to devote more time and attention and maintain the continuity of tasks (Al-Sayid and Kirkil, 2023; Wang et al., 2025). Evidence from synchronous interaction, real-time comments and collaborative discussions shows that enhanced interactive experience can bring higher quality cognitive processing and participation (Zhang et al., 2025). Based on this, this study puts forward the following assumptions:

*H2*: Interactive experience has a significant positive impact on CE.

### 3.4 Moderating role of PI on the SP–CE relationship

The cognitive benefits of social interaction need interactive channels to convey social clues and support meaning negotiation. When the interactive experience is high, social clues are easier to be perceived and responded, and the interaction is more continuous. Therefore, learners are more likely to turn social contact into content-centered questioning, argumentation and integration. The research on gamification and structured interaction shows that enhancing interaction can promote the dissemination and collaborative processing of social information, thus promoting deep cognitive activities (Wijaya et al., 2025; Mu et al., 2025). Based on this, this study puts forward the following assumptions:

*H3*: PI can positively regulate the relationship between SP and CE.

## 4 Research methods

### 4.1 Participants

This study recruited college students with online learning experience as participants. In order to ensure the validity and ethical compliance of the sample, all respondents should be at least 18 years old and sign an informed consent form before filling out the questionnaire to confirm their voluntary participation. Data were collected through the online platform of “Online Learning Experience Survey”, and an electronic questionnaire named “Online Learning Experience Survey” was distributed to college students by snowball sampling method. Participants range from freshmen to seniors with a wide range of professional backgrounds, including humanities, social sciences, natural sciences and engineering. This composition ensures the representativeness and diversity of the sample in demography. Table 1 lists the predefined sample composition and the distribution characteristics of participants.

**Table 1:** Predefined sample composition and distribution characteristics of participants.

Variables	Categories
Gender	Male, Female
Grade level	Freshman, Sophomore, Junior, Senior
Major type	Humanities, Social Sciences, Sciences, Engineering, Others
Online learning characteristics	Weekly online learning hours, years of online learning experience

The target group is college students who are currently studying or have completed full-time undergraduate courses and have at least one semester’s experience in online credit courses. Data collection adopts network platform and snowball sampling method. The initial sample is student leaders and class representatives from five comprehensive universities in different regions. They distributed the e-questionnaire links to their respective class groups and learning communities. Respondents voluntarily completed the questionnaire after informed consent.

### 4.2 Measurement instruments

In this study, a structured questionnaire was used to collect data, which included demographic information and three core psychological scales. Except for demographic variables, all items were scored by five-point Likert scale. SP is measured by nine core items in CoI Scale developed by Arbaugh et al. (2008), which cover emotional expression, open communication and group cohesion, and aim to evaluate learners’ “realism” in virtual environment. CE is evaluated by the online student participation scale proposed by Dixson (2015). The scale selects eight items reflecting deep cognitive processing, self-regulated learning strategies and cognitive efforts to capture learners’ higher-order thinking activities more accurately. PI is measured by the adapted version of the scale developed by McMillan and Hwang (2002). The scale is reduced to eight items, including two dimensions-perception of media interaction and perception of social interaction-to evaluate learners’ subjective perception of system responsiveness, fluency of interaction and effectiveness of interpersonal communication. The detailed structure and item contents of each scale are listed in Table 2.

**Table 2:** Measurement items for research variables.

Variable dimension	Item code	Item description
SP	SP1	Getting to know other learners in this course made me feel that I was part of the course.
	SP2	I was able to form clear and specific impressions of some course participants.
	SP3	Online or web-based communication was a high-quality way to engage in social interaction.
	SP4	I felt natural and comfortable when communicating online.
	SP5	I felt relaxed and at ease when participating in discussions in this course.
	SP6	I felt comfortable interacting with other learners in this course.
	SP7	Even when disagreements occurred, I could express different opinions within an atmosphere of mutual trust.
	SP8	I felt that my viewpoints received attention and recognition from other course participants.
	SP9	Online discussions helped me feel a sense of collaboration and collective learning with others.
CE	CE1	I planned and scheduled my online learning in a regular manner.
	CE2	I was generally able to keep up with the required readings or learning materials.
	CE3	Before logging in to learn again, I reviewed class notes or records to ensure my understanding of the content.
	CE4	I took careful notes while reading materials, viewing slides, or watching instructional videos.
	CE5	I listened attentively or read carefully when studying online course content.
	CE6	I actively thought about how to connect course content to my life or personal experiences.
	CE7	I attempted to apply what I learned in online courses to real-life or learning contexts.
	CE8	I genuinely hoped to master the content of this online course.
PI	PMI1	The online learning platform provided sufficient learning path options, allowing me to control my progress and content.
	PMI2	The platform responded smoothly and promptly to my actions.
	PMI3	I could easily find the information I needed on the platform.
	PMI4	The platform offered corresponding content or feedback based on my learning behaviors or preferences.
	PSI1	The online course provided convenient conditions for two-way communication with the instructor.
	PSI2	The platform supported interaction between me and other learners.
	PSI3	The discussion areas or interactive spaces allowed me to express ideas and receive responses.
	PSI4	The course design encouraged communication and exchange with other learners.

### 4.3 Data analysis methods

After the data collection is completed, this study mainly relies on SPSS (social science statistics software package) to systematically analyze the effective samples. In the data preprocessing stage, invalid answers and questionnaires with abnormal answer patterns were eliminated, and basic statistical analysis was carried out to understand the distribution characteristics of samples. Cronbach’s *α* coefficient and factor analysis were used to test the reliability and validity of each scale to ensure that all measuring tools meet the psychometric standards. On this basis, Pearson correlation analysis is carried out to explore the pairwise correlation among SP, CE and PI. The hypothesis test adopts hierarchical linear regression analysis. The nested model is constructed to test the main effect, and the interactive term is introduced to test whether PI plays a moderating role in the relationship between variables, thus revealing the potential psychological mechanism.

All data processing is completed by SPSS 27.0. Invalid answer sheets and questionnaires with abnormal or irregular answer patterns were eliminated. Hierarchical linear regression analysis was used to test the main effect and potential regulatory effect. In order to avoid multicollinearity, before constructing interactive variables, all continuous variables involved in interactive items are mean-centered (Shcheglova, 2019; Fathi et al., 2024).

## 5 Results analysis

### 5.1 Descriptive statistical results

Table 3 shows the demographic distribution of the sample. As shown in the Table 3, the gender composition is relatively balanced, accounting for 52.6% of women and 47.4% of men. This balanced ratio helps to avoid measurement bias caused by gender sample deviation. As far as the academic year is concerned, sophomores and juniors account for the majority of the participants, totaling more than 60%. Students at this stage have usually adapted to university study and have rich online learning experience, so they can make a more accurate evaluation of online interactive experience. As far as the subject background is concerned, the distribution of samples in social sciences, natural sciences, engineering and humanities is relatively uniform, and the proportion of major disciplines is between 18 and 29%. This extensive discipline coverage enhances the universality and ecological validity of the research results in different learning groups.

**Table 3:** Demographic characteristics of the sample [(A) Gender; (B) Grade Level; (C) Major Type].

Dimension	Category	Frequency (N)	Percentage (%)
Gender	Male	237	47.4
	Female	263	52.6
Grade level	Freshman (Year 1)	87	17.4
	Sophomore (Year 2)	149	29.8
	Junior (Year 3)	173	34.6
	Senior (Year 4)	91	18.2
Major type	Social Sciences	141	28.2
	Natural Sciences	115	23.0
	Engineering	110	22.0
	Humanities	94	18.8
	Others	40	8.0

Table 4 lists the descriptive statistics and normality test results of continuous variables. As shown, the average age of college students participating in the survey is 20.58 years old, which is in the early adult stage with active cognition, which is usually related to higher learning motivation and autonomous learning ability. They study online for more than 5 h a week on average, and have nearly 3 years of online learning experience, which shows that participants have developed good study habits, are familiar with digital learning platforms, and have been fully exposed to various online teaching forms. The core latent variables also show good data characteristics: the average score of SP is 3.67, and the average scores of PI and CE are 3.59, which are higher than the midpoint of the scale, indicating that participants generally have a strong sense of SP and show a high level of CE in the virtual learning environment. In addition, the absolute values of skewness and kurtosis of all variables are far less than 2, which proves that their distributions are approximately normal. The results verify the appropriateness of applying parameter statistical methods (such as correlation analysis and hierarchical regression) in subsequent hypothesis testing, and provide a solid foundation for ensuring the reliability and robustness of the research results.

**Table 4:** Descriptive statistics and normality tests for continuous variables.

Variable	Minimum	Maximum	Mean	SD	Skewness	Kurtosis
Age (years)	18	26	20.58	1.65	0.434	−0.042
Weekly Online learning hours	0.1	19.4	5.15	3.26	1.151	1.619
Online learning experience (years)	0	7.9	2.93	1.55	0.084	−0.361
SP	1.11	5.00	3.67	0.70	−0.330	−0.037
PI	1.50	5.00	3.59	0.69	−0.182	−0.334
CE	1.50	5.00	3.59	0.74	−0.115	−0.526

### 5.2 Correlation analysis

In order to ensure the robustness of the research results, this section adopts a comprehensive analysis strategy. Table 5 lists the reliability and convergence validity indicators of each measurement dimension. As shown in the table, the measurement tools used in this study show extremely high data quality. The Cronbach’s *α* coefficient of SP, PI and its sub-dimensions and CE all exceeded 0.90, and the combined reliability (CR) value also exceeded 0.90. These results show that there is excellent internal consistency among the items, which verifies the stability of the measurement concept. AVE of all potential variables is much higher than the threshold of 0.50-the AVE of CE is as high as 0.713. Combined with significant Bartlett test results and high Kaiser-Meyer-Olkin (KMO) value, these indicators confirm that the scale meets the reliability standard. They also show good convergence validity, which provides a solid psychometric basis for investigating the structural relationship between variables.

**Table 5:** Reliability and validity test results for measurement instruments.

Latent variable	No. of items	Cronbach’s α	CR	AVE	KMO	Bartlett *χ*^2^	*p*-value
SP	9	0.941	0.938	0.684	0.935	2845.12	<0.001
PI	8	0.918	0.915	0.647	0.892	2103.56	<0.001
Perceived media interactivity	4	0.889	0.885	0.658	–	–	–
Perceived social interactivity	4	0.892	0.890	0.671	–	–	–
CE	8	0.952	0.949	0.713	0.941	3012.44	<0.001

The results of correlation matrix and discriminant validity are shown in Table 6. The square root of AVE value (shown on the diagonal) is greater than the correlation coefficient between the corresponding variable and other variables, which shows that the discrimination validity is good. From the perspective of correlation strength, SP and CE showed a strong positive correlation (r = 0.764), indicating that higher SP is often accompanied by deeper CE. PI also shows a high correlation with SP (r = 0.713), which provides direct statistical support for the view that frequent and high-quality interactive experience is the basic mechanism for constructing SP in virtual environment.

**Table 6:** Correlation matrix and discriminant validity.

Variable	1	2	3	4	5
1. SP	(0.827)				
2. CE	0.764**	(0.845)			
3. PI	0.713**	0.672**	(0.804)		
4. Perceived media interactivity	0.635**	0.601**	0.908**	(0.811)	
5. Perceived social interactivity	0.654**	0.615**	0.915**	0.663**	(0.822)

**p < 0.01; values in parentheses represent the square root of AVE. Values greater than the correlations with other variables indicate good discriminant validity.

### 5.3 Hypothesis testing

In this study, hierarchical linear regression is used to strictly test the main effect and regulatory effect. In order to eliminate the potential multicollinearity, both the SP and the regulating variable (PI) are mean-centered before constructing the interaction item. Model 1 includes gender, age and other demographic variables as control variables; Model 2 adds independent variable SP and regulating variable PI; finally, model 3 includes the interaction term of SP × PI.

Table 7 lists the complete results of stratified regression analysis. It can be seen that demographic control variables such as gender and age have weak explanatory power to CE, and Model 1 as a whole has no statistical significance. When the core independent variable is introduced, the explanatory power of model 2 is significantly improved, and the adjusted R is 0.611. SP has a strong positive predictive effect on CE, the standardized regression coefficient is 0.582, and PI also has a significant positive predictive effect on CE. In model 3, after adding the interaction term, R did not increase significantly, and the coefficient of interaction term was only 0.035, which did not reach statistical significance. This shows that PI is not a regulating variable and will not change the intensity of SP effect. Combined with the high correlation observed before, PI is more likely to be a necessary prerequisite variable for SP formation.

**Table 7:** Hierarchical regression results for CE.

Predictor	Model 1		Model 2		Model 3	
	*β*	*t*	*β*	*t*	*β*	*t*
(Constant)		6.214***		1.542		1.635
Control variables						
Gender (1 = Male)	−0.045	−0.982	0.015	0.512	0.016	0.545
Age	0.053	1.154	−0.012	−0.421	−0.013	−0.456
Grade	0.021	0.488	0.018	0.635	0.019	0.668
Online learning	0.082	1.821	0.015	0.518	0.017	0.589
Experience	−0.031	−0.695	−0.008	−0.286	−0.009	−0.324
Independent variable						
SP			0.582*	14.531	0.578*	14.356
Moderator						
PI			0.257*	6.412	0.251*	6.185
Interaction term						
SP × PI					0.035	1.165
Model fit						
$R^{2}$	0.015		0.617		0.618	
Adjusted $R^{2}$	0.005		0.611		0.611	
$�R^{2}$	0.015		0.602		0.001	
*F*	1.485		109.39***		95.74***	
Δ*F*	1.485		389.15***		1.358	

*Dependent variable = CE; ***p < 0.001, **p < 0.01, *p* < 0.05; all variables were mean-centered prior to analysis.

Table 8 lists the multiple collinearity diagnostic indexes of the regression model. All independent variables and regulating variables are mean-centered before constructing interactive items, which effectively reduces false multicollinearity. The tolerance of all the predicted variables remained at about 0.49, and the variance expansion factor (VIF) was about 2.03, which was far below the commonly accepted threshold of 5, which confirmed the stability and reliability of the regression estimation. Regulation analysis shows that PI does not significantly change the influence of SP on CE. In view of the cross-sectional design adopted in this study, it is impossible to draw strict time causal inference. The observed high correlation between PI and SP is more likely to indicate that in the virtual learning environment, these two concepts coexist as complementary psychological states, which together provide a supporting background for higher-level cognitive activities, rather than a simple boundary condition.

**Table 8:** Multicollinearity diagnostics for the regression model.

Model path	Unstandardized coefficient	Std. error	Standardized coefficient	*t*	Tolerance	VIF
SP → CE	0.619	0.043	0.582	14.531	0.491	2.035
PI→CE	0.314	0.049	0.257	6.412	0.491	2.038
SP × PI→CE	0.042	0.036	0.035	1.165	0.982	1.018

Table 9 shows a simple slope analysis of the influence of SP on CE at different PI levels. When PI is low, SP has a significant positive effect on CE (*β* = 0.590, *p* < 0.001), and the 95% confidence interval [0.478, 0.702] does not contain zero. With the increase of PI to the average level and high level, the influence increased slightly, reaching 0.619 and 0.648 respectively, and it was still highly significant. However, the *Δ* r of the interaction term is only 0.001, *F*(1, 492) = 1.358, and *p* = 0.244, indicating that the difference of slope at different PI levels is not statistically significant. These results show that the influence of SP on CE is stable regardless of the level of PI, which proves that PI plays a role as a stable background factor rather than a regulatory factor, which also explains why hypothesis 3 is not supported.

**Table 9:** Simple slope analysis of SP on CE at different levels of PI.

PI level	Effect	Std. error	*t*	*p*	95% CI
Low (−1 SD)	0.590	0.057	10.352	0.000	[0.478, 0.702]
Mean	0.619	0.043	14.356	0.000	[0.535, 0.703]
High (+1 SD)	0.648	0.052	12.461	0.000	[0.546, 0.750]
Effect difference test	Δ*R*^2^ = 0.001	*F*(1, 492) = 1.358	*p* = 0.244	(Interaction not significant)	

Low, mean − 1 SD; High = mean + 1 SD.

## 6 Discussion

This study confirms that SP is the core psychological mechanism that drives learners to carry out deep CE in online learning environment. The data show that learners’ willingness to participate in higher-level cognitive activities is significantly enhanced when they perceive strong interpersonal relationships and realism in virtual space. This finding is consistent with the research results of Li and Ye (2025), who pointed out that psychological isolation in online environment is the main obstacle to deep learning, and SP can effectively alleviate this sense of alienation and promote learners to shift from shallow browsing to deep knowledge construction. Different from previous studies that mainly focused on the function of technology platform, this study emphasized the transformation of psychological perception, which further supported the view of Liao et al. (2023) that social cues in the technology environment can activate learners’ metacognitive monitoring, thus enhancing the depth of cognitive processing.

With regard to the role of PI, this study clarifies the function of interpersonal perception as a “necessary premise” rather than a “boundary regulator” by denying the moderating hypothesis and confirming its high correlation with SP. The results of this study extend the research of Zhong et al. (2022), who mainly investigated the direct influence of interactive behavior on learning achievement and satisfaction. The results of this study show that the interactive experience must first be transformed into the psychological state of SP before it can affect the cognitive results. This explains why just increasing the frequency of interaction does not always improve the learning effect-CE (cognitive experience) will only appear when interaction successfully creates a common feeling of “being together.” Fung et al. (2025) discussed the influence of different types of interaction on learning motivation; on this basis, this study further confirms that interpersonal interaction and human-computer interaction are essentially fertile ground for SP formation. The research results correct the previous hypothesis that interaction has a moderating effect, and show that once SP is established, its positive impact on CE is steady and direct no matter how the interaction type changes.

PI does not significantly regulate the relationship between SP and CE, which reveals the more fundamental “basic-core” logic in online learning environment, rather than the originally assumed “enhancement-regulation” logic. The data denies the hypothesis that interactive technology only plays a catalytic role. On the contrary, they support the existence of threshold effect. In the virtual learning space, PI is more like a “health factor” in herzberg’s two-factor theory than a real “incentive factor.” When the availability or response speed of interactive function is lower than a certain threshold, technical barriers will hinder the transmission of social clues, thus preventing the formation of SP. However, once the interaction reaches a smooth and stable level, adding more interactive functions or improving the response speed of the system will not continuously enhance its impact on the social CE. This result shows that technical interactivity is a necessary way to the psychological presence, but it is not the ultimate driving force. When learners cross the threshold of “technical transparency,” their attention will shift from operating interface to processing content and sensing other people’s emotions. At this stage, the depth of social emotional experience no longer depends on the width or speed of interactive channels, but on the quality of social emotional information flowing in them. Interaction lays the foundation for social telepresence, but it cannot control the transformation from social telepresence to social emotional experience.

An in-depth study of PI and its basic functions reveals the complementary relationship between human-computer media interaction and human-to-human social interaction. In the current digital teaching practice, functions such as real-time comment, real-time audio connection and collaborative whiteboard are not only channels for information transmission, but also amplifiers for social signals. Media interaction ensures the accessibility of information and smooth operation experience, reduces the sense of alienation brought by the interface, and gives learners a sense of control over the system. In contrast, social interaction ensures the transmission of emotions. Through likes, emoticons and instant feedback, it injects warmth and human touch into the original data-oriented environment.

Both types of interaction are indispensable for building SP. Without social interaction, a smooth interface will form an information island; However, if the strong social intention is restricted by the delayed or cumbersome interface, it will lead to the frustration of learners and eventually lead to the collapse of social interaction. Therefore, high-quality online teaching design must achieve the best balance between technical availability and social support. PI is a necessary prerequisite for social interaction. Its core value lies in giving learners a digital certificate of “being there” in virtual space. It transforms the identity on the screen into a real and vivid individual, and activates a deeper cognitive process in the process.

The educational significance of these findings is that both platform developers and front-line educators should deeply integrate technical usability and emotional support. In the design of platform interface, interactive function should go beyond simple function stacking and turn to emotion-centered design logic. Micro-interactive modules that can convey nonverbal cues, such as real-time emotional feedback symbols, dynamic collaborative cursors and common visual indicators, should be integrated to reduce the cognitive load of learners in the process of system operation. On the teaching level, teachers should establish clear online interaction norms, carry out ice-breaking activities and heterogeneous cooperation groups at the beginning of the course, and create a virtual community with high psychological security. For the daily management of the discussion forum, the study should regularly publish open-ended questions that can lead to cognitive conflicts, and give instant and personalized text or voice feedback to high-quality interactive contributions. Building strong interpersonal trust in this way can encourage learners to continue deep collaborative learning.

## 7 Conclusion

Overall, this study confirmed that SP is the core psychological factor to predict learners’ deep CE in online learning environment. PI is closely related to social participation, which together constitute a potential supporting environment to drive cognitive participation. This study expounds the parallel and synergistic effect of personal participation and social participation in predicting cognitive participation from the theoretical level, corrects the view that technical interaction only plays a simple regulatory role in previous literature, and enriches the interpretation of interaction paths between key interaction variables. Due to the cross-sectional nature of the survey data, this study cannot fully capture the evolution trajectory or time causality between variables. Future research should adopt longitudinal design and integrate multi-modal objective behavior data (such as platform usage log, mouse trajectory or eye tracking) to more accurately describe the dynamic fluctuation of social psychological state during the course and its long-term impact on cognitive participation.

## Data Availability

The original contributions presented in the study are included in the article/supplementary material, further inquiries can be directed to the corresponding author.
